# Phenotypic and genetic variation in the response of chickens to *Eimeria tenella* induced coccidiosis

**DOI:** 10.1186/s12711-018-0433-7

**Published:** 2018-11-21

**Authors:** Kay Boulton, Matthew J. Nolan, Zhiguang Wu, Androniki Psifidi, Valentina Riggio, Kimberley Harman, Stephen C. Bishop, Pete Kaiser, Mitchell S. Abrahamsen, Rachel Hawken, Kellie A. Watson, Fiona M. Tomley, Damer P. Blake, David A. Hume

**Affiliations:** 1 The Roslin Institute, University of Edinburgh, Easter Bush, Midlothian, UK; 2 Department of Pathobiology and Population Sciences, Royal Veterinary College, University of London, Hatfield, UK; 3 Department of Clinical Sciences and Services, Royal Veterinary College, University of London, Hatfield, UK; 4 Cobb-Vantress Inc., PO Box 1030, Siloam Springs, AR USA; 5 Mater Research Institute, University of Queensland, Brisbane, St. Lucia, QLD, Brisbane, Australia

## Abstract

**Background:**

Coccidiosis is a major contributor to losses in poultry production. With emerging constraints on the use of in-feed prophylactic anticoccidial drugs and the relatively high costs of effective vaccines, there are commercial incentives to breed chickens with greater resistance to this important production disease. To identify phenotypic biomarkers that are associated with the production impacts of coccidiosis, and to assess their covariance and heritability, 942 Cobb500 commercial broilers were subjected to a defined challenge with *Eimeria tenella* (Houghton). Three traits were measured: weight gain (WG) during the period of infection, caecal lesion score (CLS) *post mortem*, and the level of a serum biomarker of intestinal inflammation, i.e. circulating interleukin 10 (IL-10), measured at the height of the infection.

**Results:**

Phenotypic analysis of the challenged chicken cohort revealed a significant positive correlation between CLS and IL-10, with significant negative correlations of both these traits with WG. Eigenanalysis of phenotypic covariances between measured traits revealed three distinct eigenvectors. Trait weightings of the first eigenvector, (EV1, eigenvalue = 59%), were biologically interpreted as representing a response of birds that were susceptible to infection, with low WG, high CLS and high IL-10. Similarly, the second eigenvector represented infection resilience/resistance (EV2, 22%; high WG, low CLS and high IL-10), and the third eigenvector tolerance (EV3, 19%; high WG, high CLS and low IL-10), respectively. Genome-wide association studies (GWAS) identified two SNPs that were associated with WG at the suggestive level.

**Conclusions:**

Eigenanalysis separated the phenotypic impact of a defined challenge with *E. tenella* on WG, caecal inflammation/pathology, and production of IL-10 into three major eigenvectors, indicating that the susceptibility-resistance axis is not a single continuous quantitative trait. The SNPs identified by the GWAS for body weight were located in close proximity to two genes that are involved in innate immunity (*FAM96B* and *RRAD*).

**Electronic supplementary material:**

The online version of this article (10.1186/s12711-018-0433-7) contains supplementary material, which is available to authorized users.

## Background

In chickens, seven species of *Eimeria* (Apicomplexa, Coccidia) are responsible for the debilitating and sometimes fatal disease coccidiosis that is estimated to cost the international poultry industry around US$3 billion per year, mainly due to reduced productivity and the cost of preventive measures [1, 2]. Current control of coccidiosis relies on the prophylactic use of synthetic or fermented ionophore anticoccidial drugs and on vaccination with formulations of live wild-type or attenuated parasites [3–5]. Use of some anticoccidial drugs has been curtailed by legislation in many countries, while the costs and limited production capacity of live attenuated vaccines compromise their utility in broiler flocks [6]. Thus, identification of traits that may contribute towards selective breeding of chickens to control the consequences of coccidiosis is of great importance to industry.

**Fig. 1:**
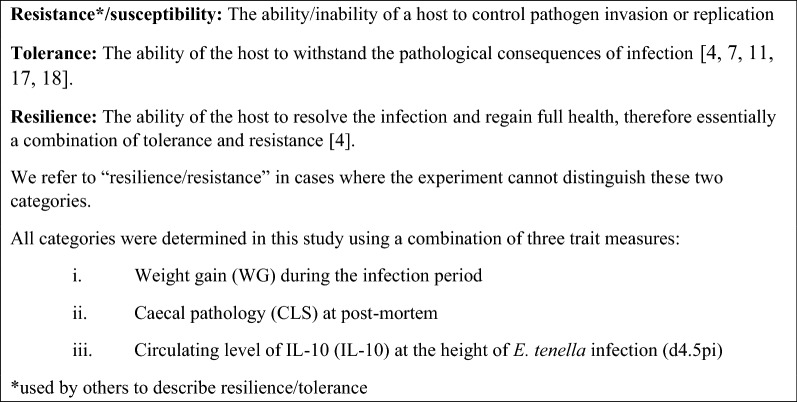
Definitions of immune categories used

In principle, the impact of *Eimeria* infection could be mitigated by selecting chickens that limit their infectious load by being refractory to parasite infection (resistance), by tolerating the consequences of infection (tolerance), or by recovering from pathological consequences of infection sufficiently quickly to maintain their growth and body condition (resilience). Resistance, resilience and tolerance to disease are distinct, individual-specific traits that are likely to have a different genetic basis [7, 8]. However, phenotypic distinction between these three traits is difficult without a longitudinal measurement of pathogen load [9–11]. Nevertheless, there is evidence for genetic differences among chicken populations in their response to *Eimeria* parasitism. For example, some native chicken breeds, such as the Egyptian Fayoumi, appear to tolerate the pathological impacts of *Eimeria* infection [12, 13], while distinct inbred lines of White Leghorn chickens support variable levels of parasite replication by different *Eimeria* species [14]. Biological pathways that may be indicative of resistance to *Eimeria maxima* infection have been noted in modern commercial broilers [15, 16]. One genome-wide association study (GWAS) [16] alluded to the mechanisms of resistance to *E. maxima* including primary innate immune response, tissue repair, and proliferation. Definitions of the immune categories used in this study are presented in Fig. 1 [4, 7, 11, 17, 18].

*Eimeria* species that infect chickens have life cycles of 4 to 14 days in vivo that include self-limiting phases of asexual (schizogony) and sexual (gametogony) reproduction. *E. tenella* causes haemorrhagic coccidiosis in chickens, with morbidity and mortality being highest during maturation and rupture of the second-generation schizonts in the caeca, starting from − 108 h (4.5 days) post-infection [19]. The caecal lesions caused are sometimes exacerbated by bacterial co-infection and *E. tenella* is therefore associated with local induction of a wide range of pro- and anti-inflammatory cytokines [20]. Selection of chickens with intrinsically high or low levels of pro-inflammatory cytokines and chemokines in response to *E. tenella* (i.e. IL-6, CXCLi2, and CCLi2) has revealed an association of innate immune response with resistance to the pathology that is associated with coccidial infections [21].

Induction of the anti-inflammatory and immunosuppressive cytokine, interleukin-10 (IL-10), was first characterised in the spleen and intestine of chickens of inbred White Leghorn lines infected with *E. maxima* [22]. Both constitutive and inducible levels of IL-10 mRNA were higher in “disease-susceptible” than in “disease-resistant” lines. Later, enhanced expression of IL-10 mRNA was shown in the caeca of broilers infected with *E. tenella* [23]. Monoclonal antibodies and an ELISA (enzyme-linked immunosorbent assay) test to detect IL-10 in chicken blood were recently developed and, as in mice, IL-10 was shown to be an inhibitor of both innate and acquired immune responses, with a marked elevation in chickens challenged with *E. tenella* [24]. This finding agrees with the proposed specific function of IL-10 in immune regulation in the intestine, based upon spontaneous colitis that develops in IL-10 knockout mice [25].

Mechanisms of innate immunity have been alluded to in many previous studies that investigated genetic resistance to coccidiosis [10, 13, 16, 19, 22, 26, 27]. However, those exploring response to *E. tenella* were performed using either microsatellite or low-density single nucleotide polymorphism (SNP) panels to map quantitative trait loci (QTL) in inbred chicken lines [13, 27]. A previous large-scale GWAS using the Cobb500 investigated a different *spp.* (*E. maxima*) with a commercially available SNP panel [17]. In this study, our aim was to investigate the relationship between circulating IL-10 (IL-10) as a quantitative trait and two standard phenotypic measures of *E. tenella* infection: weight gain (WG) and caecal lesion score (CLS), to assess the heritability of each trait and to identify candidate genetic markers that impact the outcome of coccidial challenge. We hypothesized a positive relationship of circulating IL-10 with disease pathology (CLS), and negative relationships of both these traits with WG, and the identification of significant SNPs that correlate with these traits.

## Methods

### Animals and management

In this study, 1200 1-day old infectious bronchitis-vaccinated (Nobilis IB H120, MSD Animal Health, Milton Keynes, UK) commercial four-way crossbred Cobb500 broiler chickens (*Gallus gallus domesticus*) were acquired from a major UK supplier. These comprised three replicate intakes of 375 (intake_1_, September 2016), 376 (intake_2_, October 2016) and 391 (intake_3_, November 2016) individuals at the time of infection. At the conclusion of the trial, 1142 animals were available for sampling. For each intake, chicks were initially housed together in a coccidia-free environment at a DEFRA (Department for Environment Food & Rural Affairs) approved stocking density of 34 kg/m^2^ (anticipated end weight) under a UK Home Office project licence. Up to day 10, chickens received a starter feed that contained the anticoccidial drug Maxiban^®^ (Elanco; Greenfield, Indiana, USA; 31.25 g/kg feed). On day 11, an anticoccidial-free grower diet was introduced, and dust-extracted pinewood shaving bedding (StableBed, Essex, UK) was replaced to diminish the risk that anticoccidial residues in the bedding affected future experimental procedures. Consistent with standard commercial broiler rearing management, the grower diet was replaced with finisher pellets on day 25 (Target Feeds Ltd, Shropshire, UK). The light:dark ratio and temperature were changed according to commercial broiler rearing practice.

### Experimental design

**Fig. 2:**
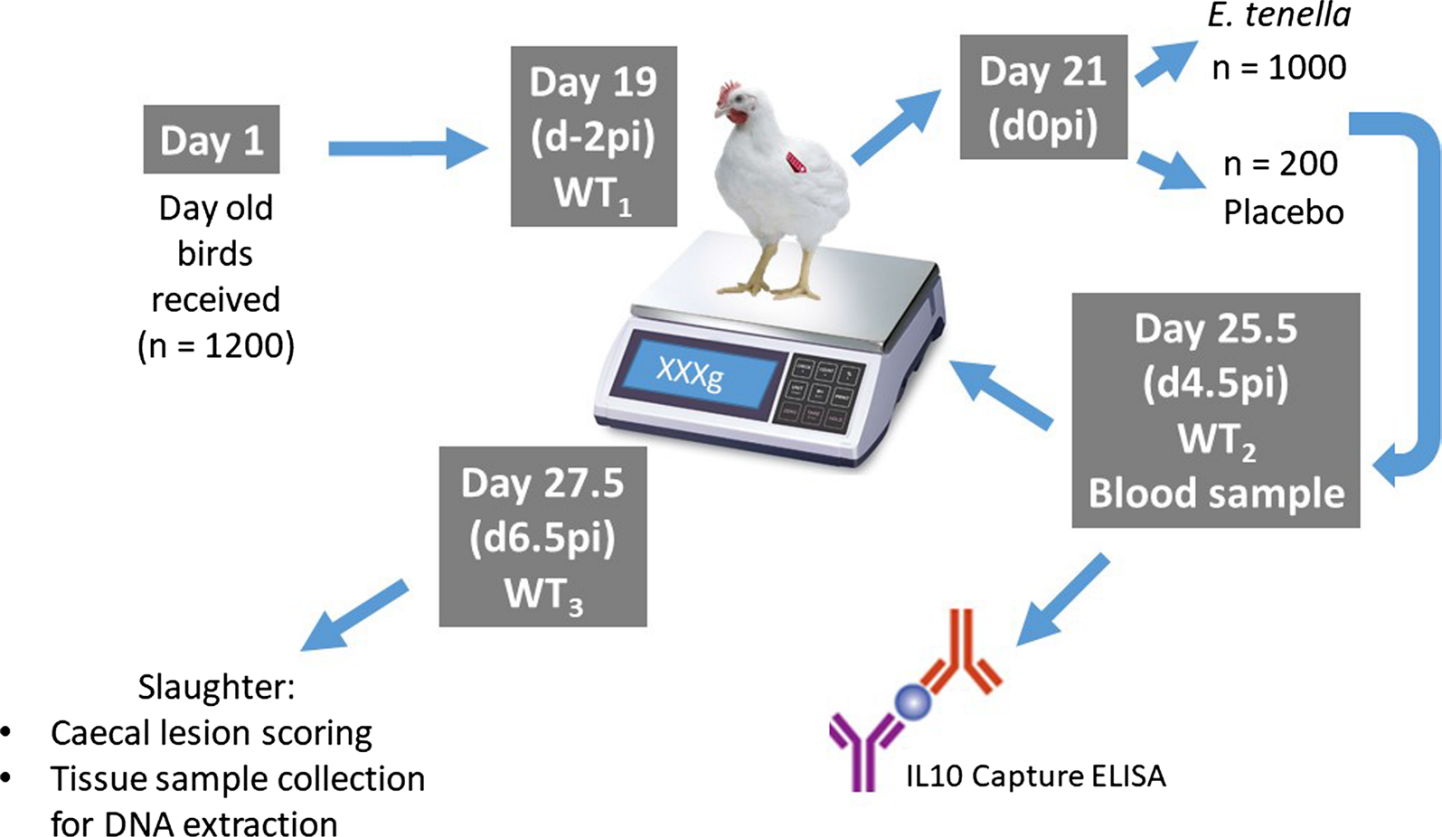
Experimental design

The design of the study is summarised in Fig. 2. To facilitate animal management, at day 19 each chick intake was randomly divided into two separately housed equally-sized groups, referred to as replicates ‘a’ and ‘b’, that were handled on consecutive days (day_1_ and day_2_). At d-2 post-infection (pi), birds were wing tagged for individual identification and weighed (WT_1_, WT for body weight) to the nearest 0.01 g using digital scales (Kern CXB 3K0.2, KERN & SOHN GmbH, Balingen, Germany). Following WT_1_, birds were randomly assigned to control (intake_1_, n = 100; intake_2_, n = 50; and intake_3_, n = 50) or infection (intake_1_, n = 275; intake_2_, n = 326; and intake_3_, n = 341) within-replicate groups. On d0pi, control birds received a unique 1.0 mL inoculum of DNase/RNase free H_2_O. The remaining birds were infected via oral gavage with a unique 1.0 mL inoculation of *E. tenella* (Houghton strain) sporulated oocysts. Individuals in intakes_1, 3_ received 42,500 2-month old oocysts, while those in intake_2_ received 40,000 1-month old oocysts. Oocyst sporulation was verified by light microscopy. Birds assigned to replicate ‘a’ received inoculations on day 21, while those assigned to replicate ‘b’ were inoculated on day 22, i.e. replicate ‘b’ gained an additional day of infection-free growth. Infected and control birds were housed in separate rooms, thus allowing both sufficient space to meet commercial environmental conditions and minimising the risk of accidental infection of the control birds with *Eimeria* parasites.

At d4.5pi (108 h), all birds were weighed (WT_2_) and a 0.5-mL blood sample was collected from the brachial vein. Blood was immediately transferred to a 1.5 mL microcentrifuge tube (Sigma-Aldrich, Dorset, UK), allowed to clot overnight at 4 °C, and centrifuged at 2000 × *g*/10 min. Then, the serum was aspirated into a sterile microcentrifuge tube and stored at − 20 °C, for IL-10 assay at a later date. Circulating IL-10 was measured by Capture ELISA according to [24], using ROS-AV164 and biotinylated ROS-AV163, as capture and detection antibodies, respectively.

At d6.5pi (156 h), birds were culled by cervical dislocation and weighed *post*-*mortem* (WT_3_). Both caeca were removed and scored individually for lesion damage [19] by the same experienced operator throughout the entire study. For DNA isolation, a 1-cm section of the base of each caecal pair was transferred to a 7 mL Sterilin™ bijou tube (ThermoFisher Scientific, Waltham, MA, USA) that contained 5–10 volumes of RNA*later*^®^ at room temperature (Life Technologies, Carlsbad, CA, USA), according to the manufacturer’s instructions. The DNeasy^®^ Blood and Tissue Kit (QIAGEN, Hilden, Germany) was used to isolate total genomic DNA (gDNA) from each caecal tissue sample, following the manufacturer’s instructions. DNA extracts were stored at − 20 °C. A total of 942 DNA samples were available from infected chickens and were genotyped by GeneSeek, a Neogen Company [28] using a proprietary 62K SNP array (Cobb-Vantress, Arkansas, USA).

### Phenotypic statistical analyses

Three phenotypic traits were measured: body weight (WT, g) at time-points d2pi (WT_1_), d4.5pi (WT_2_) and d6.5pi (WT_3_); circulating IL-10 (pg/mL) at d4.5pi, and caecal lesion score (CLS, on a scale of 0-4) *post*-*mortem* (d6.5pi). The derived trait, weight gain (WG = WT_3_–WT_1_) was also considered. Since the pathology of the two caeca from a bird may differ, CLS were allocated per bird according to the higher score for each caecal pair. A natural log-transformation was applied to IL-10 for it to conform to a Gaussian distribution. The same transformation did not improve the negative distribution skew of CLS, thus these data were not adjusted. Formal testing of differences in weight traits and in the three phenotypic traits of interest (WG, CLS and IL-10) between control and infected birds was carried out in ASReml 4.0 [29] using a simple linear univariate model Eq. (1) to generate predicted means:

1 $${\mathbf{y}} = {\mathbf{Xb}} + {\varvec{\upepsilon}} ,$$

where ${\mathbf{y}}$ is the vector of observations, ${\mathbf{X}}$ is the design matrix relating fixed effects to observations, ${\mathbf{b}}$ is the vector of fixed effects, and **ε** is the vector of residual effects. Fixed effects included intake (a three-level factor), trial replicate (a two-level management within intake factor, accounting for the 1-day handling age difference: ‘a’ and ‘b’), sex (male or female), and all significant interaction terms. Weight at d-2pi (WT_1_) was fitted as a covariate in all analyses of weight traits except WT_1_. To ascertain the most parsimonious model for each phenotypic trait, fixed effects were formally tested and removed from the individual trait model if their significance was above the 5% threshold based on a conditional Wald F-test.

All three traits of interest were subsequently rescaled (mean-centred/standard deviation) to adjust for differences in units of measurements. Using ASReml 4.0 [29] and referring to the univariate models for the assignment of significant fixed effects to each trait, a multivariate linear model was used to estimate the phenotypic variance–covariance matrix (${\mathbf{P}}$) among traits, i.e. no genetic effect was fitted at this stage. Likelihood ratio tests (LRT) were used to determine the significance of trait (co)variances by comparing the full multiple trait model with a model that constrained trait covariances to zero. This approach avoided differences in environmental variance (or measurement error) that can generate spurious support for correlation significance.

An eigen decomposition was applied to **P** and the percentage of variance and trait loadings for each eigenvector (EV) was derived. Trait loadings for each EV were plotted on a histogram to facilitate interpretation of the between-trait correlations, following [30].

### Genetic statistical analyses

Genotyping the 942 infected individuals resulted in 62,732 SNPs located on 28 autosomes, the W and Z sex chromosomes, and four unplaced scaffolds. GenABEL [31] was used to construct a genetic relationship matrix (GRM) based on the SNP genotypes and used in a univariate linear mixed model for each trait (using ASReml 4.0 [29]) to estimate variance components and genetic parameters Eq. (2):

2 $${\mathbf{y}} = {\mathbf{Xb}} + {\mathbf{Za}} + {\varvec{\upepsilon}} ,$$

where ${\mathbf{y}}$, ${\mathbf{X}}$, ${\mathbf{b}}$ and **ε** are defined as in Eq. (1), ${\mathbf{a}}$ is the vector of additive genetic effects, and ${\mathbf{Z}}$ is a design matrix relating random effects to observations. Weight at d-2pi was included as a covariate for WG trait. Heritability of each trait was calculated as the ratio of additive genetic and phenotypic variance. Genetic correlations between traits were estimated by bivariate analyses using Model (2).

For GWAS, quality control removed individuals and SNPs that failed according to the following criteria: minor allele frequency lower than 0.05 to distinguish common polymorphisms from rare variants); call rate lower than 90%; cut off individual call-rate lower than 90%; identity by state threshold less than 1; Hardy–Weinberg equilibrium *P* $\le$ 10^−6^. Thus, 921 individuals and 46,836 SNPs remained for further analyses. Classical multidimensional scaling of the SNP genotypes was conducted to confirm the genetic homogeneity of the sample before analysis. Since no obvious substructure was detected (see Additional file 1: Figure S1), we did not consider it in further analyses.

A separate GWAS was performed for each trait in GenABEL [32], fitting sex, intake, and replicate as fixed effects and the GRM as a polygenic effect to correct for genetic relationships. The −log_10_ *P* value from the Wald test was corrected for possible inflation of lambda [32]. Thresholds for genome-wide (*P* ≤ 0.05) and suggestive (i.e. accounting for one false discovery per genome-scan; significance levels were calculated using the Bonferroni correction for multiple-testing, i.e. −Log_10_ $\times$ (0.05/total number of SNPs) and −Log_10_ $\times$ (1.0/total number of SNPs), respectively. To determine the effects associated with significant SNPs identified in the GWAS, single-marker association analyses were carried out using ASReml v 4.1, as in Eq. (2). Predicted trait values for each SNP genotype, represented by ${\text{AA}}$, ${\text{BB}}$ and ${\text{AB}}$, and SNP allele frequencies $p$ and $q$ were used to estimate the additive (a = (AA − BB)/2) and dominance (d = AB − [(AA + BB)/2] effects and the variance at the SNP ($V_{{{\text{A}}\_{\text{SNP}}}} = [2pq\left( {a + d\left( {q - p} \right)} \right)^{2}$], assuming Hardy–Weinberg equilibrium genotype frequencies. The heritability of each SNP was then calculated as $V_{{{\text{A}}\_{\text{SNP}}}}$ divided by the $V_{\text{P}}$ of the trait.

To identify candidate genes, the significant SNPs identified by GWAS were converted from the *Gallus*-*gallus*-4.0 to the *Gallus*-*gallus*-5.0 assembly using the LiftOver tool [33] Then, genes located 250 kb up- and down-stream were annotated using the data mining tool BioMart [34].

## Results

Data from the six replicates (2 replicates for 3 intakes) were pooled according to the assigned infection status. The numbers of birds falling into each category are summarised in Table S1 (see Additional file 2: Table S1).

### Phenotypic analyses

#### Body weight and weight gain

**Fig. 3:**
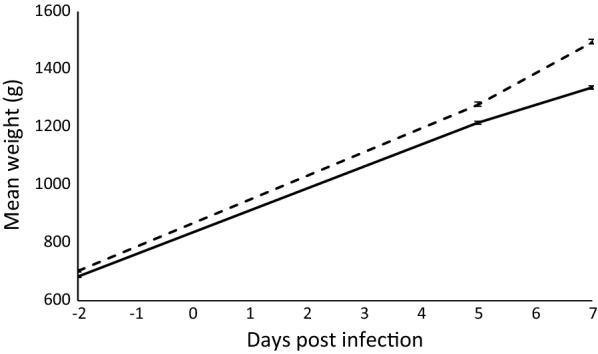
Mean weight (g) of control (n = 200, dashed-line) and infected (n = 942, solid-line) birds throughout the trial period, with standard error bars. See also (Additional file 2: Table S2) for full details

**Fig. 4:**
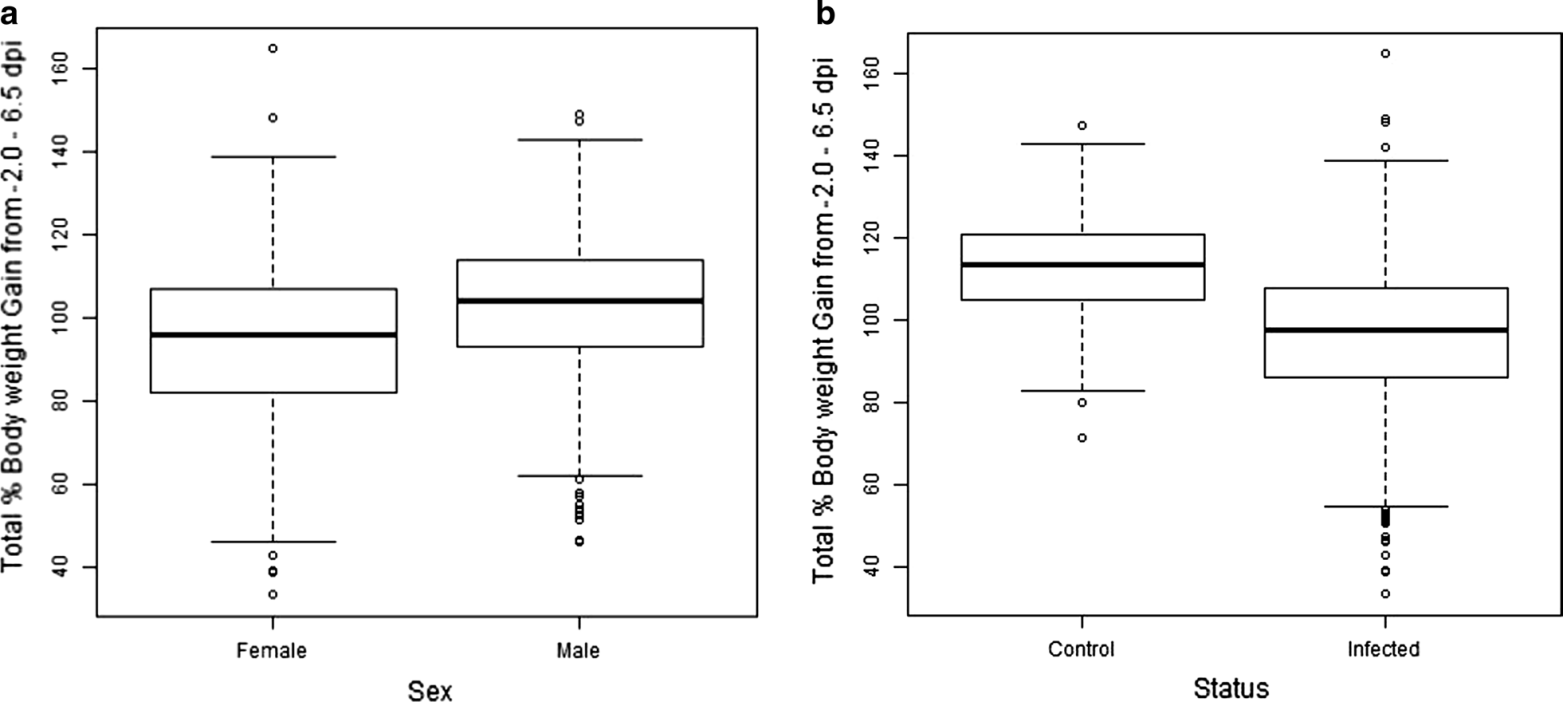
Box plot of body weight for **a** control and infected birds and **b** the two sexes at d6.5pi

Significant differences were found between the unadjusted weights of control and infected birds data (Fig. 3). Univariate analysis of mean-adjusted phenotypic data revealed significant differences (*P* < 0.001) between control and infected chickens for body weight at d-2pi (WT_1_), d4.5pi (WT_2_), and d6.5pi (WT_3_) and for weight gain (Fig. 4a; see Additional file 3: Table S2). Mean weights and mean weight gains were also significantly (*P* < 0.001) different between the three experimental intakes (see Additional file 3: Table S2). Males were significantly heavier than females for all measures (*P* < 0.001; Fig. 4b; see Additional file 3: Table S2).

#### Relationships between WG, CLS, and serum IL-10

**Fig. 5:**
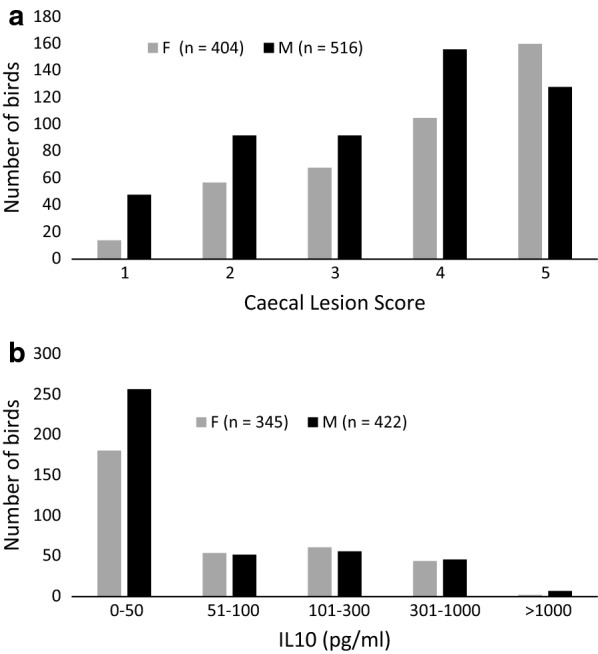
Distribution of **a** caecal lesion score and **b** IL-10 for male (M) and female (F) infected birds

No caecal pathology was detected in the control chickens (CLS score = 0). Phenotypic variance in CLS was observed in infected chickens, with the full spectrum of scores (0–4) observed across both sexes (Fig. 5a). Both intake and replicate explained some of the variance in CLS (*P* < 0.001; see Additional file 3: Table S2).

Serum IL-10 concentrations in the control chickens were below the assay limit of detection but were elevated to varying extents in birds challenged with *Eimeria*, as previously described [22]. The difference between male and female infected chickens in the production of IL-10 was not significant (*P* = 0.54; Fig. 5b; and (see Additional file 3: Table S2).

**Fig. 6:**
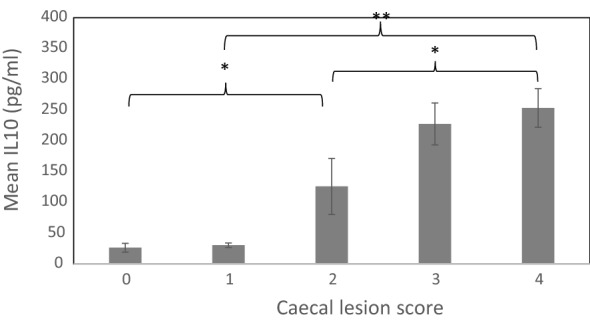
Visualisation of the relationship between caecal lesion score and IL-10 (pg/ml) in infected birds. *P < 0.05; **P < 0.001

A linear model multivariate analysis was used to estimate phenotypic correlations ($r_{\text{P}}$) between the three measured traits (*P *< 0.001, Table 1). WG was negatively correlated with both CLS (− 0.37 ± 0.03) and IL-10 (− 0.35 ± 0.03), while the correlation between CLS and IL-10 was positive (0.39 ± 0.03; Fig. 6). Overall, birds with high CLS tended to have high IL-10 and low WG.

**Table 1:** Estimates of the phenotypic variances, covariances, and correlations produced by multivariate mixed model analysis

	WG	CLS	IL-10
WG	**0.679 (0.032)**	− 0.366 (0.029)	− 0.350 (0.032)
CLS	− *0.291 (0.028)*	**0.928 (0.043)**	0.393 (0.030)
IL-10	− *0.275 (0.030)*	*0.361 (0.035)*	**0.903 (0.046)**

Between-trait covariances are below the diagonal (italics), phenotypic trait variances are on the diagonal (bold), with between-trait correlations above the diagonal. Measured traits are weight gain (WG), caecal lesion score (CLS) and serum interleukin 10 (IL-10). Standard errors (± SE) are presented in parentheses

Using the univariate linear mixed model (Eq. 2), genetic variance and heritability were evident for WG and CLS but not for IL-10. Only the heritability estimate for WG was significantly different from zero (0.19 ± 0.09; Table 2). The multivariate linear mixed model and the bivariate models that included IL-10 as a response variable did converge, likely due to the absence of detectable genetic variance for IL-10. The estimate of the genetic correlation between WG and CLS was negative and significant (− 0.94 ± 0.32).

**Table 2:** Suggestively significant SNPs with their GalGal 5.0 location (chromosome and position in base pairs) and *P* values for each of the measured phenotypic traits

SNP	Location Chr: bp	*P* value	$h^{2}$ trait (SE)	$V_{\text{P}}$ trait (SE)	Minor allele frequency	$h^{2}$ SNP	$a$ (SE)	*P* ($a$)	$d$ (SE)	*P* ($d$)
WG_1	11:4,244,594	1.34 × 10^−5^	0.191 (0.093)	0.683 (0.033)	0.43	0.026	21.05 (4.99)	< 0.001	− 6.37 (6.37)	0.241
WG_2	11:11,383,252	1.49 × 10^−5^			0.42	0.026	22.42 (5.00)	< 0.001	3.46 (6.46)	0.346
CLS_1	12:17,993,212	4.62 × 10^−5^	0.071 (0.083)	0.930 (0.044)	0.33	0.001	0.19 (0.08)	0.019	0.054 (0.094)	0.337
IL-10_1	4:7,575,042	2.763 × 10^−5^	0 (0)	0.921 (0.047)	0.28	–	–	–	–	–

Univariate linear mixed models performed using Eq. 2 (ASReml 4.0), provided the trait phenotypic variance ($V_{\text{P}}$; means-adjusted) and trait heritability ($h^{2}$) with standard errors (SE). The non-means-centered additive ($a$) and dominance ($d$) effects of the SNPs are presented with their significance values (*P*). SNP heritabilities ($h^{2}$ SNP) were calculated as additive variance of the SNP divided by $V_{\text{P}}$ of the trait. Major ($p$) and minor ($q$) SNP allele frequencies are also presented. Non-estimable values for the IL-10 SNP are indicated (−)

### Eigen analysis

**Fig. 7:**
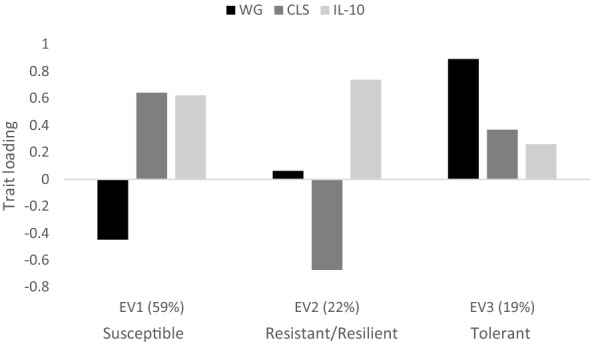
Eigenvector decomposition of the phenotypic covariance matrix. Vector loadings for the three measured traits [weight gain (WG), caecal lesion score (CLS) and serum interleukin-10 (IL-10)] are presented in standard deviation units on the y-axis. The percentage of variance explained by each eigen vector (EV) is in brackets on the x-axis

The modest phenotypic correlations between traits were investigated further using an eigen decomposition of the phenotypic covariance (Fig. 7). Trait weightings of the first eigenvector, (EV1, eigenvalue = 59%), were low WG, high CLS and high IL-10, and likely represent a susceptible spectrum, as defined in Fig. 1. In these birds, it is assumed that the low WG is a consequence of the parasite-induced caecal pathology. The second and third eigenvectors (EV2, 22%; high WG, low CLS and high IL-10, and EV3, 19%, high WG, high CLS and low IL-10) separate birds that may be considered as resistant/resilient or tolerant, respectively. The birds with high values for EV2 may produce an effective immune response, including production of IL-10, that either prevents pathogen invasion/replication or mitigates the pathology, i.e. they are either resistant or resilient; these alternatives cannot be distinguished. The birds with high values for EV3 gain weight despite caecal pathology and the lack of an innate immune response (low IL-10), i.e. they are phenotypically tolerant. Plotting the trait loadings for each EV, as in Fig. 7, enabled a visualisation of the multivariate distribution of partly overlapping immune categories which include susceptible, resilient/resistant and tolerant.

### Genotypic analysis

#### Association analysis

**Fig. 8:**
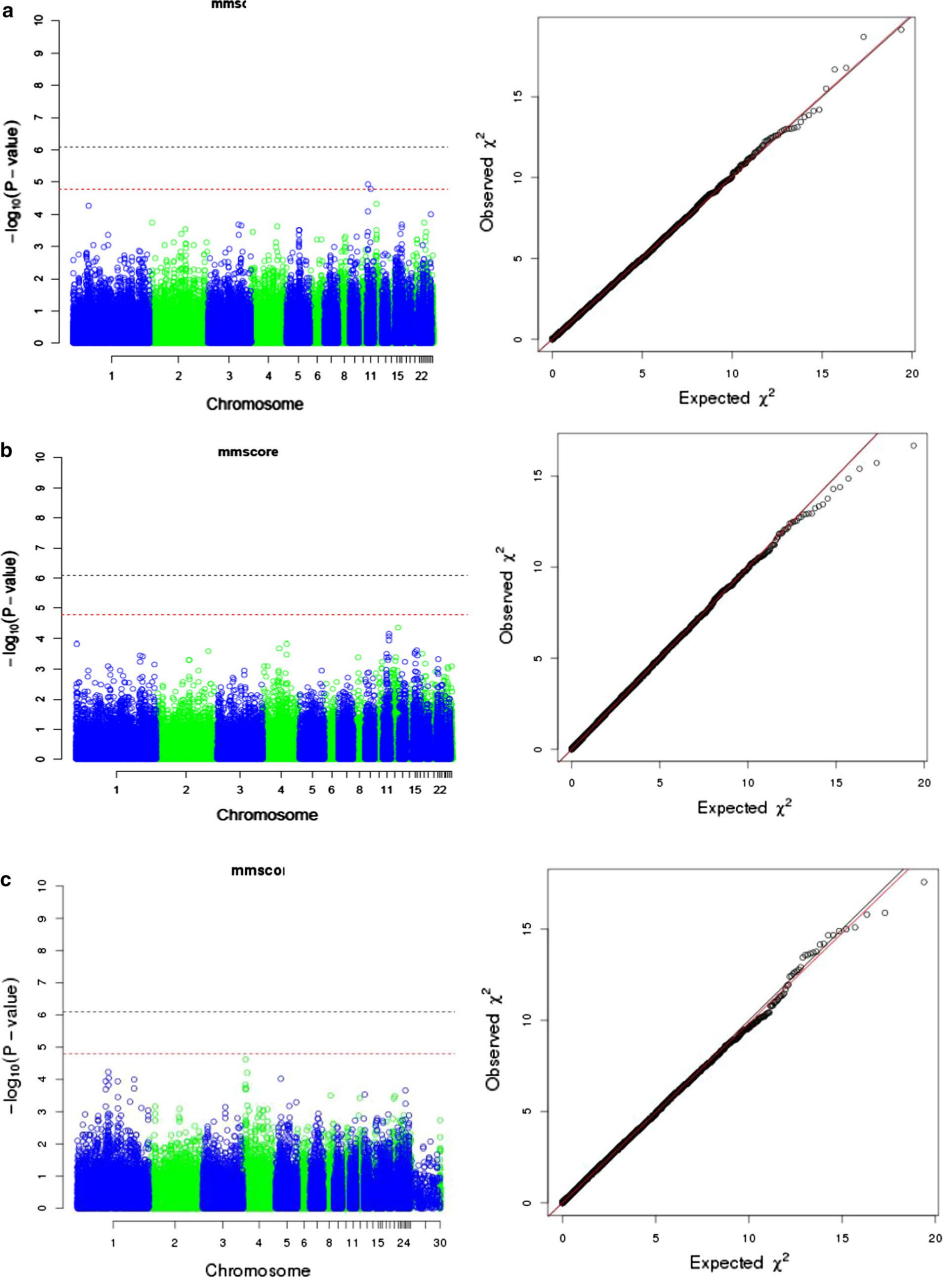
Manhattan and corresponding QQ plots from the GWAS for: **a** weight gain; **b** caecal lesion score; **c** serum IL-10. The log_10_ *P* value is plotted for each SNP on the relevant chromosome (x-axis). Bonferroni corrected thresholds were set as *P* ≤ 1.1×10^−6^ and *P* ≤ 2.1×10^−5^ for genome-wide (*P* ≤ 0.05) and suggestive (i.e. one false discovery per genome-scan) levels, corresponding to −log_10_ *P* values of 5.97 (blue) and 4.67 (red) lines

Following quality control of the data, 921 of the 942 infected birds were available to detect significant associations between SNPs and the analysed traits. After Bonferroni correction for multiple testing and adjustment for the test inflation factor, two suggestive SNPs were identified for the derived trait, WG, during the period of infection. Both were located on chromosome 11 (Table 2; Fig. 8a). These SNPs were 7.76 Mb apart (i.e. at opposite ends of the microchromosome) and not in linkage disequilibrium (LD). The strongest association with CLS was observed for a SNP on chromosome 12 (Table 2; Fig. 8b). However, after correction, this association was not significant. Lastly, one SNP was associated with IL-10 level at the height of infection, on chromosome 4 (Table 2; Fig. 8c). Given the lack of detectable genetic variance for this trait, this is likely a false positive. Corresponding quantile–quantile (q–q) plots for the three GWAS analyses are in Fig. 6a, b and c. Small additive effects (*P* < 0.003) were estimated for the significant SNPs, (Table 2). Estimates of dominance effects were not significant. The effect of the significant SNPs were also found to be significant (*P* < 0.001) in the univariate linear mixed models.

## Discussion

The objective of this study was to estimate the variance and investigate the genomic architecture of chicken response to *E. tenella* infection, using the Cobb500 commercial broiler. GWAS of the three analysed phenotypic traits revealed the presence of suggestive significant SNPs for body weight gain (WG) but not for caecal lesions or IL-10. Significant genetic variance and heritability was detected for WG only. For CLS both these estimates had large standard errors. We could not detect genetic variance for IL-10, and the reasons for this may be complex. In addition, it was clear that IL-10 production was not strongly correlated with caecal pathology. A future study will focus on the relationship between quantified pathogen load and circulating IL-10 to determine whether IL-10 production is an independently heritable trait. Based on estimates of variance and the extensive heterozygosity of the four-way crossbred population, it is now clear that our study was underpowered to identify the genetic control of the response to *Eimeria* challenge. The Cobb500 genotype was selected deliberately to establish whether substantial phenotypic variation exists in response of commercial broiler chickens following a defined infection with *E. tenella*, the cause of caecal coccidiosis. The phenotypic results indicate that this is clearly the case. We were, however, unable to estimate heritabilities that were significantly different from zero, but it is unclear if this was a result of the four-way cross or that variance in CLS and IL-10 has no underlying genetic basis. Further investigation is required to determine if the assays used here could be applied to pedigree lines to select for the desired phenotype(s).

Innate immune response is highly conserved across chordates and IL-10 is known to be critical for resistance to gastrointestinal parasite infections in mice [35]. As noted above, selection for enhanced production of pro-inflammatory cytokines has been reported to increase resistance to *E. tenella* [21] and increased expression of these cytokines in the intestinal mucosa of birds infected with *E. acervulina* has been documented e.g. [36]. However, the published studies have assessed neither the utility of these responses as biomarkers, nor their variance on a population scale in commercial chicken lines. Here, we focused on the anti-inflammatory cytokine IL-10 that was previously reported to be significantly elevated in the serum of *E. tenella* challenged birds [24]. Although the function of IL-10 in host defence in *Eimeria* infection is unclear, there is evidence that it contributes to the pathology in some manner, as the use of oral antibodies against IL-10 has been reported to mitigate the growth rate suppression that occurs in broiler chickens infected with *Eimeria* [37, 38]. Another reason for our focus on IL-10 was to identify a quantifiable phenotype in the early stage of disease challenge that could provide a biomarker for genetic selection.

Quantification of individual disease severity was assessed *post*-*mortem* by scoring caecal lesions, with males exhibiting, on average, less damage compared to females. Interestingly, a previous study [39] found that significantly more male embryos survived infection with *E. tenella*, suggesting that innate coccidiosis resistance differs between sexes. In line with our hypothesis that birds with high WG have, on average, lower CLS and IL-10, the estimated phenotypic correlations of WG with CLS and IL-10 were negative (− 0.37 ± 0.03 and − 0.35 ± 0.03, respectively), indicating that birds failed to thrive due to, at least in part, the direct effects of *E. tenella* infection. These phenotypic correlation estimates are consistent with other reports e.g. [40] (− 0.14), [41] (only a negative direction is indicated) and [42]. The positive phenotypic correlation between CLS and IL-10 (0.39 ± 0.03) was consistent with the relationship between susceptibility and high IL-10 mRNA production in mucosa among inbred chicken lines [22]. A weakness of our study is that circulating IL-10 was measured at a single time-point only. In that sense, the 0.39 phenotypic correlation with caecal lesion score is remarkable.

We concentrated on the use of eigenvalue decomposition of the phenotypic variance–covariance matrix among traits, rather than principal components because the eigenvector trait loadings provide population evidence for variance in the immune categories described in Fig. 1 [43]. If phenotypic immune response was a simple susceptible/resistant axis, then all the variance should be explained by the first eigenvector. However, this was not the case, with 41% of the variance shared between eigenvectors 2 and 3.

Trait loadings for EV1 reflected the estimated between-trait correlation directions that are indicative of susceptibility: i.e. the ability/inability of a host to control pathogen invasion or replication, resulting in a lack of weight gain, a high level of pathology and a strong response from the innate immune system. In the most affected birds, a high level of circulating IL-10 is likely to be the consequence of pathology and local immune responses, and is consistent with the variation observed among inbred birds that differ in resistance [22]. Certainly, *E. tenella* infection impairs normal caecal function such as fermentation of complex carbohydrates, nitrogen absorption, and water retention. In field situations, haemorrhagic disease induced by *E. tenella* causes diarrhoea, leaves birds weak and dehydrated with reduced appetites and the damaged intestine susceptible to secondary infection by opportunistic intestinal bacteria. EV2 displayed no loading for WG, and a negative loading on CLS, thus indicating that part of the variation in CLS is independent of WG. Nevertheless, the high IL-10 loading for EV2 indicates that there was a strong response to the pathogen, and we interpret this vector to be indicative of resistance/resilience. Finally, the EV3 trait loadings displayed severe pathology while simultaneously retaining growth performance although the loading on IL-10 was very low, thus our biological interpretation is that this eigenvector represents a tolerant phenotype.

We speculate that elevation of IL-10 could induce systemic immunosuppression that may underlie the observed negative correlation of serum antibody titres against bacterial and viral pathogens with parasite load in field studies of native birds in Africa [42]. In mammalian species, there is strong evidence that polymorphisms in the *IL*-*10* and *IL*-*10 receptor* genes are responsible for susceptibility to gastrointestinal inflammatory disease [44].

As noted above, the population used in this study may not be ideal for a genetic analysis due to its four-way cross construction. Other studies that reported significant SNP associations with WG and *Eimeria* infection used larger populations of the same broiler [16] and/or different population structures [13, 15, 45–47]. Two suggestive SNPs on chromosome 11 were associated with body weight. Putative candidate genes *FAM96B* (*family with sequence similarity 96 member B*) and *RRAD* (*Ras related glycolysis inhibitor and calcium channel regulator*) for these SNPs are associated with gastrointestinal and metabolic diseases, and with the development of the digestive system and disorder networks, [48–50].

A separate study that used Cobb500 broilers infected with the small intestine parasite *E. maxima*, found several significant QTL for disease resistance but none of these were identified in our study [16]. This could be due to one or more of the many differences between these two studies, including those relating to the pathology and immune responses induced by *E maxima* versus *E. tenella,* the phenotypes measured, sample size, sex ratio, age of birds at infection, and density of SNP information available. Keeping the birds for a further 7 days may have elucidated the difference between resistance and resilience by enabling measurement of the extent of recovery, but it would have precluded collection of CLS that must be scored between 5 and 7 days post-infection [19] since pathological lesions resolve after this time. Two previous QTL mapping studies on resistance to *E. tenella* infection used a Fayoumi $\times$ White Leghorn intercross rather than a commercial line, measured phenotypes that, apart from WG, could not be compared to our study, [13, 27]. Only one of these studies [27] located QTL on chromosomes 4 and 11 but none of these were located in similar regions or were linked to phenotypes of the suggestive SNPs found our study.

Resilient birds are attractive to industry since they are able to overcome disease, acquire immunity, and return to their original healthy condition. However, pathogens can mutate rapidly to overcome host resistance that can lead to a selective advantage of mutants in resistant individuals. It is unclear whether tolerance would be a desirable production phenotype without information on other commercially important traits such as feed conversion efficiency. Genetic change of the pathogen may be smaller if it can complete its life cycle in tolerant birds [51], but birds that can tolerate disease could also be “super-shedders” that drive environmental oocyst numbers upward. IL-10 production may be beneficial or detrimental to the host. Other studies have suggested that IL-10 is directly involved in the reduced body weight gain observed in *Eimeria*-infected birds [37, 38]. The existence of birds that produce high levels of IL-10 but gain weight normally argues against such a direct role.

We anticipate extending our studies to pedigree lines where a simpler genetic relationship matrix can be established, and to natural infection, over a longer time course, where we can also measure the trajectory of circulating IL-10, pathogen load, shedding, and feed conversion efficiency. There is also a need to test whether circulating IL-10 is specific to the parasite infection or could provide a more general indication of intestinal health.

## Conclusions

We have confirmed the existence of substantial variance for weight gain in birds infected with *E. tenella*, a trait that is used traditionally as a measure of resilience/resistance to coccidiosis caused by this pathogen. Our data indicate that breeding for increased weight gain under challenge alone would not distinguish between resilience/resistance and tolerance. Eigen analysis of the phenotypic variance–covariance structure among weight gain, caecal inflammation/pathology and production of IL-10 separated the phenotypic impact of the defined challenge with *E. tenella* into three major eigenvectors, indicating that the susceptibility-resistance axis in this population is complex. We were unable to demonstrate significant genetic basis for the variation in CLS and IL-10. However, suggestive SNPs identified by the GWAS for body weight were located in close proximity with two genes known to be involved in innate immunity (*FAM96B* and *RRAD*).

## Additional files

**Additional file 1: Figure S1.** Plot of population substructure, as explored by classical multidimensional scaling using GenABEL.

**Additional file 2: Table S1.** Number of birds used in this 3 × 2 × 2 × 2 design by category:-Intake (1, 2, 3), disease status (control or infected), replicate (a, b) and sex.

**Additional file 3: Table S2.** Univariate linear model analyses performed in ASReml 4.0 were used to predict the means (PM) of the fixed effects on each of the measured traits, with standard errors (± SE) and significance (P value).
