# Supplementary material for: Phenotypic and genetic variation in the response of chickens to Eimeria tenella induced coccidiosis
Source: Genet Sel Evol. 2018 Nov 21;50:63. doi: 10.1186/s12711-018-0433-7 (PMC6249784; doi:10.1186/s12711-018-0433-7)

**Additional File 1**

**Figure S1**

Plot of population substructure, as explored by classical multidimensional scaling (MDS) using GenABEL.


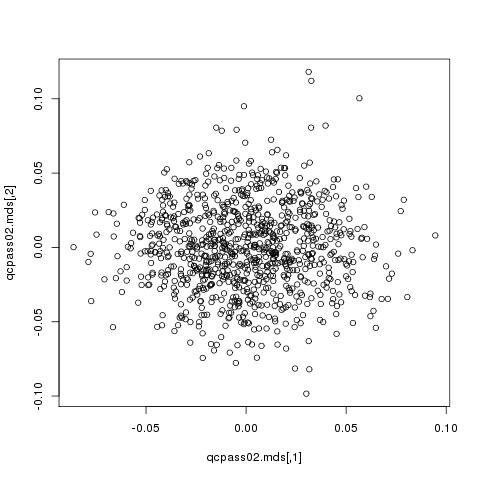

Supplement: Supplementary file 1 — Additional file 1: Figure S1. Plot of population substructure, as explored by classical multidimensional scaling using GenABEL. [file 12711_2018_433_MOESM1_ESM.docx]
