# Supplementary material for: Phenotypic and genetic variation in the response of chickens to Eimeria tenella induced coccidiosis
Source: Genet Sel Evol. 2018 Nov 21;50:63. doi: 10.1186/s12711-018-0433-7 (PMC6249784; doi:10.1186/s12711-018-0433-7)
Supplement: Supplementary file 2 — Additional file 2: Table S1. Number of birds used in this 3 × 2 × 2 × 2 design by category:-Intake (1, 2, 3), disease status (control or infected), replicate (a, b) and sex. [file 12711_2018_433_MOESM2_ESM.docx]

**Additional File 2**

**Table S1** The number of birds used in this 3x 2 x 2 x 2 designed study by category:-Intake (1, 2, 3), disease status (control or infected), replicate (a, b) and sex.

| Disease Status | Control | | | | Infected | | | |
| --- | --- | --- | --- | --- | --- | --- | --- | --- |
| Sex | Male | | Female | | Male | | Female | |
| Replicate | a | b | a | b | a | b | a | b |
| Intake 1 | 41 | 38 | 9 | 12 | 87 | 86 | 61 | 41 |
| 2 | 9 | 10 | 16 | 15 | 92 | 85 | 84 | 65 |
| 3 | 12 | 12 | 13 | 13 | 101 | 76 | 74 | 90 |
| Sub total | 62 | 60 | 38 | 40 | 280 | 247 | 219 | 196 |
| Total | 122 | | 78 | | 527 | | 415 | |
